# Pulmonary Consumption, Successfully Treated with Naphtha

**Published:** 1843-10

**Authors:** 


### Art. XVIII.

*Pulmonary Consumption, successfully treated with Naphtha.* By John Hastings, m.d., Senior Physician to the Blenheim Street Free Dispensary.—*London,* 1843. 8vo, pp. 120.

We have risen from the perusal of this book with varied feelings of a most unpleasant kind; and if that fertile source of inspiration acknowledged by the poet in the well-known verse,

"Si natura negat facit INDIGNATIO versum,"

were permitted to the scientific critic, assuredly the present article would be both copious and severe. But reason, not passion, must guide the pen of the reviewer, whose office ought ever to be that of a judge and never that of a satirist; we will, therefore, repress all emotional feelings, and sit down calmly to dispense justice.

We say JUSTICE; according to our solemn conviction of what is right and true, after complete investigation of the premises, and without affection or favour, on the one hand, and without prejudice or malice on the other. And we are proud to say that this is a merit which this Journal may boldly claim. In witness of its right so to do, we refer fearlessly to every Number of our now-long series, and to every article of every Number; and we will add, that it is a merit that it shall not forfeit so long as the hand that has hitherto directed its course shall continue to rule and guide it. In making this statement, we do not pretend to maintain that all our opinions have been correct and all our judgments just; to do so would be to assert a claim incompatible with the imperfection of human things; but we do maintain that where we have erred we have never done so wilfully, nor yet through carelessness or a laxity of principle almost as criminal as conscious corruption. Would that the past and present state of medical criticism in this country had made the character now claimed for this Journal in no respect singular or peculiar! But, alas, when we see published, week after week, quarter after quarter, accounts of medical books so utterly at variance with their real character, we feel ourselves warranted in the adoption of a tone which, in other circumstances, would be presumptuous. In charging some of our contemporaries with this delinquency, we entirely acquit the editors of voluntary and conscious injustice; we merely accuse them of a culpable carelessness, and of indulging in the silly and vain hope of reaping where they have not sowed. So long as it can be said of any journal that it will admit into its pages an author's criticism of his own book, or a friend's criticism of his friend's book, or a publisher's puff of *his* book, or, to speak in general terms, so long as gratuitous contributions from suspicious or incompetent hands are permitted to figure as literary adjudications, so long shall we continue to denounce much of the medical criticism of our time; so long shall we look upon our own labours with conscious satisfaction, nor feel the

—Sume superbiam
Quæsitam meritis———

of the poet, to be a mandate suggestive of reproach instead of honour.

These reflections have arisen in our mind while meditating on the character of the judgment which justice compels us to pronounce on the book now before us, and anticipating, in imagination, the very opposite verdict that will probably be returned in other courts of criticism, where perchance laxer and more good-natured judges may preside over more facile juries. Be this as it may, we, at least, shall not shrink from the discharge of our duty, however painful, nor wittingly incur the penalty implied in the once obscure but now immortal apophthegm,

"Judex damnatur cum nocens absolvitur."

It is doubly painful to us, both from national and personal considerations, to be called on to notice a book like that of Dr. John Hastings, (we earnestly entreat the reader not to confound the author of this book with the honorable and honoured founder of the Provincial Medical Association, Dr. Charles Hastings of Worcester,) just after rising from the task of reviewing the immortal work of M. Louis; and the more so when we reflect that we have had to do like justice on so many kindred productions of our countrymen, on the same subject, during the last few years. It was only in our Number for April last, that we found ourselves called on, while noticing *two* new books on consumption, to make use of the following somewhat severe expressions:

"But this matter grows too serious to be treated triflingly. For, though we are unwilling to acquiesce in the common notion that the learned writers produce these books with the simple view of advertising themselves, and for our own parts feel contented to regard them as the offspring solely of a somewhat impertinent vanity, yet productions of the sort are not as harmless as they might appear. They in truth lend their little aid in supporting and giving vigour to the monstrous system of fraud practised by the 'consumption-curing' doctors on the too gullible multitude. Viewed in this aspect they are mischievous in the extreme; though redolent of the very refuse of Paternoster Row, their influence upon the sufferers from consumption will not be the less active. Though utterly destitute of logic, as of evidence of even moderate acquaintance on the part of the writers with the principles of precise medicine, they will not fail, because they hold out hopes of cure, to lure many a victim to the toils set for all whose pockets are sufficiently well lined to make them worth the catching." (Vol. XV. p. 540.)

And we are bound to add, in justice to the gentlemen here inculpated, that the volume of Dr. John Hastings ("in the lowest deep a lower deep") is, in many respects, more deserving of reprobation than theirs. In the first place, it is written in a much worse style; secondly, it betrays still greater ignorance of precise medicine and all the rules of logic; thirdly, it is much more calculated to impose upon the public and the more ignorant and inexperienced members of the profession, by the boldness of its tone, the long array of alleged cures which it displays, and, above all, by the specious manner in which it has, for the first time, enlisted auscultation as a false witness in the cause of empiricism. It is this last enormity which chiefly roused our indignation in perusing the pages of Dr. John Hastings. We felt, while reading his cases, as if the staff on which we were accustomed to lean in all our difficulties, had not only snapt asunder beneath our weight, but had wounded us as we fell; our most cherished medicines seemed converted into poisons; we started as if the benevolent genius which had for so many years been leading us on to truth, had suddenly been transformed into a malignant demon to lure us to destruction; it was like seething a kid in its mother's milk.*

The work of Dr. John Hastings, although professedly written to magnify the virtues of naphtha in consumption, contains, of course, all the usual display of materials which the public could desiderate in a treatise on so popular a malady; accordingly, we have separate chapters on the causes, symptoms, complication, pathology, diagnosis, and treatment of consumption. Of these we shall only say, in the mass, that with the exception of a single observation of a few words on the subject of "a globule" in tubercle "hitherto unnoticed" (p. 35)—and which observation is obviously, like the rest of the book, a mistake—they do not contain one particle of information which is not to be found in the works in everybody's hands, while they are disfigured by innumerable omissions, oversights, and mistakes, and exhibit a style of composition discreditable to any man who has received even an ordinary English education, and disgraceful to one who boasts the highest honours of our learned profession. Some few of these philological delinquencies we give in a note below;†

* In reading that part of Dr. John Hastings's book which relates to diagnosis, we are forcibly reminded of a caution addressed to young auscultators by one of the earlier labourers in this field, in a very imperfect book, but one, it is believed, of much use at the time of its publication; and as the caution, even at the present day, when physical diagnosis has made such wonderful progress, seems still needed, we shall offer no apology for here transcribing a part of it:

"It is highly proper and necessary that it should be generally understood, that the practice of auscultation, although easily acquired by care and attention, is not to be acquired (in such a degree of perfection as to be useful) without these, and without considerable experience. And I would advise every one to be extremely cautious in drawing from his explorations any conclusions which are to influence the treatment of diseases, until he has been able to convince himself thoroughly of the correctness of these by a good deal of experience, and, if possible, by the unerring testimony of dissection.

"The reason of this caution will be obvious on considering the relative characters of the physical, and the common or sympathetic diagnostics. In general we depend so little on any *one* symptom of a disease, that we seldom risk any momentous treatment, or hazard a decided prognosis on it; and, therefore, it is not a matter of very great consequence, practically, whether we are right or wrong respecting its supposed import in any individual case. It is very different, however, with many of the indications furnished by mediate auscultation or percussion. To such persons as admit their authority, these carry with them the conviction of almost physical demonstration; and it is impossible not to yield to opinions founded on such a basis, assent of a very different kind from that which follows the contemplation of a mere sympathetic symptom. We may deem lightly of a quick pulse, or a hurried respiration, or an acute pain, because we know that all these may accompany an affection of the most temporary kind, and of no danger; but when we know that the fleshy sound, or absence of respiration, over one side of the chest, can only arise from a great organic change, it is impossible that we can regard such a sign but as one of the highest consequence, and as worthy to determine our prognosis and direct our practice." (Original Cases, &c. by John Forbes, M.D.—London, 1824. Preface, pp. xxiii-iv.)

† It would be too serious a task to expose all the peculiarities of style contained in the pages of Dr. John Hastings; to do so we would need to reprint his book, and our philological zeal does not go so far as this; but the following short list of specimens, taken at random from the volume, will sufficiently enlighten our readers as to the precision and clearness of the style, and the grammatical and classical knowledge of the "Senior Physician to the Blenheim Street Free Dispensary:"

"...... at no period of life *are* either sex exempt." (p. 3.)

"*Preventitive* remedy." (*Ib.*)

"Frightful as are the results, they fall far short in amount of misery *with* those of consumption." (*Ib.*)

"The most common of the various *incitements to the propagation of tubercle* is the hereditary cause." (p. 10.)

"Some individuals........blind themselves to their own condition, *by refusing their*

but we have not time to dwell on them: our business, at present, is with the matter not the form of the book—with the facts or alleged facts, which it presents to us in proof of the power of naphtha to cure consumption.

We have no right, *à priori*, to conclude that naphtha or any other unknown substance may not possess the happy distinction of being remedial of phthisis; although from the failure of tar, creosote, and other analogous substances, we might feel disposed, at first, to doubt the probability of this. And we give Dr. John Hastings all due credit for having been the means of introducing this medicament to our notice in relation to consumption. From some of the statements made by him in detailing the cases, we are disposed to hope that it may be found useful in catarrhal and other chronic affections of the air-passages; and we, further, recommend its being tried in these and also in cases of true phthisis, both in the early and later stages, in the manner prescribed by him. It is *possible*, at least, that naphtha may be a cure for consumption, though Dr. John Hastings may have failed to show that it is so.

The original part of this volume consists in the details of thirty-seven cases of pectoral affections, all regarded by the author as examples of genuine phthisis, all treated by naphtha, and *all*, or nearly all, CURED by it, in a space of time varying (to speak in round numbers) from one month to four months!

"From the very *first moment*" [how marvellously rapid is the operation of this agent and how quick the perceptions of the author!] "From the very first moment I employed naphtha in pulmonary consumption up to the present time, it has been so successful in my hands, that I have no doubt it will be found, upon careful

---

*consent to the well-marked symptoms* of the disease; while others in complaining are subjects of ridicule from their blooming cheeks............and thus *the disease progresses* to an advanced stage *before seeking* medical aid." (p. 16.)

"Flatulence and pain *exists* at the pit of the stomach after meals, which in females *is* accompanied," &c. (p. 17.)

"...... after the carbonic acid and water *has* been given off ..." (p. 27.)

"The tubercles ............... begin to soften in their *centre, which gradually extends* to the circumference." (p. 28.)

"The cough and difficulty of breathing *is*," &c. (p. 34.)

"Cheerfulness of mind and *calmness of the nervous organisation* are indispensable." (p. 44.)

"...... the cough, expectoration, and difficulty of breathing *was* very much improved." (p. 67.)

"The breakfast *hour* should be nine o'clock, and *consist* of milk," &c. (p. 43.)

"...... an evident *change* for the worse ensued, which was quickly *removed*," &c. (p. 100.)

"The *catamenia was* correct as to time." (p. 62.)

"The *catamenia was* regular." (p. 69.)

"The *catamenia was* irregular." (p. 74.)

"The *catamenia was* regular." (p. 79.)

"The *catamenia was* healthy." (p. 81.)

"The *catamenia was* irregular." (p. 82.)

"The *catamenia was* natural." (p. 86.)

"The *catamenia was* healthy." (p. 91.)

We have made these numerous citations to show that this *singular* reading of *catamenia* is not a mere typographical error. We fear some of the following come within the same category; but we hope the printer is the sole delinquent:

"And *sub-crepetant* râles, which the French call the *tempest sound*." (p. 34.)

Stethoscope is everywhere spelt *stethescope*, except in two or three instances.

*Prostrate* gland. (p. 24.)

Vis medicatrix *natura*. (p 29.)

*Sub-crepetant*. Passim.

and judicious use, to be *little less than a specific* in the earlier stages of the disease." (p. 8.)

"Single-handed, if I may be allowed the use of the expression, *it has cured pulmonary consumption in almost every case in which it has hitherto been used,* when the disease has been treated *in an early stage.* And from what I have more recently observed, although I do not consider myself justified at present to publish *it,* [what?] I am most sanguine that *even in the latter stages* of the disease *a restoration of health may generally be calculated upon.*" (p. 120.)

Such general statements as these, however remarkable, coming from a quarter hitherto unknown and consequently of no authority, would make but little impression on the minds of those who know phthisis by long and melancholy experience, or who have overlived the kindred marvels once worked in utopian medicine by digitalis, hydrocyanic acid, &c. &c. Unsupported by facts, or alleged facts, they would be utterly disregarded by men of experience, as merely yet another brood of those vain visions which, in all times, have beset the brains of incipient doctors. But when we have these statements ostensibly *borne out* by the details of the cases of thirty-seven men and women, with their names and addresses blazoned in a book,—men and women, too, all alive and well, with lungs now as sound and sonorous as a bell, although, but a few weeks or months ago, with chests as dull as a board from tubercles,—the thing becomes of much more serious import and demands close investigation.

What, then, are the "facts" which Dr. John Hastings has recorded in his book—and with which, if all the men and women of England are not soon made acquainted, it is assuredly their own fault for shutting their eyes to the gentle intimations given of its existence and the nature of its cheering contents, in every journal of the day? The "facts" are these:

Thirty-two women and four men* came to Dr. John Hastings, labouring under pectoral affections of various duration, from a few weeks to some years, and presenting, with scarcely an exception and with remarkable uniformity, the following very serious combination of *symptoms:* cough, dyspnea, copious expectoration, profuse night sweats, emaciation. Four had had hemoptysis. The nature of the expectoration is scarcely ever mentioned, and the state of the pulse only three or four times. A large proportion of the patients are stated to be of consumptive families. *Every one* of these patients, in addition to these symptoms, afforded to Dr. John Hastings auscultatory *signs* of the most formidable import: viz. *dulness over a part or the whole upper regions of the chest,* absence of the respiratory murmur in the dull spaces, or change of its natural character to that of feeble, harsh, &c. &c. In no less a number, we think, than twenty-seven out of the thirty-seven cases, the dulness is represented as existing not only *on both sides but over the upper portion of the chest generally;* in ten cases, or thereabouts, the dulness is confined to one side. In about one half of the cases the sounds of the heart are stated to be abnormally audible over the dull parts; and the respiration is recorded as *puerile* on the sound side in almost all the cases where the dulness existed only on one side. Supra-clavicular or sub-clavicular depressions are stated to exist in several cases.

It is evident that if we admit the validity of the evidence of disease here afforded to us, and implied by these symptoms and physical signs, we must concur in opinion with Dr. John Hastings, that he had to deal

* We exclude, for the present, Case Twenty-three, that of a patient in the last stage.

with serious structural disease of the lungs; in short, with pulmonary consumption in an advanced stage of its progress. And yet what is the result of his treatment of these very cases?

Under the use of naphtha (in doses varying from ten to twenty, thirty, or forty drops, thrice a day) and of naphtha alone (for in general no other means seem to have been used), ALL *are cured;* the cough, expectoration, sweats, &c., disappearing for the most part within a few weeks, and the physical signs indicating structural change, vanishing shortly after!!

That we may avoid all possibility of exaggeration in an affair of so much importance, we will here put down the precise periods within which the complete cure was effected, as evinced by the disappearance of all the physical signs in addition to the common pectoral symptoms which had, in general, taken their departure long before: and, although we might be justified by the statements and obvious inferences of the author, in classing *all* the cases as examples of *perfect cure*; still, as in a certain proportion of them (eight out of the thirty-six) it is not positively said that there were no symptoms or signs left at the period of closing the report, we shall divide the cases into two classes; viz. (1) cases in which all symptoms and physical signs had disappeared, and the cure was therefore perfect; and (2) cases in which, although the patient was regarded as cured, some trifling relics of functional or structural change still remained.

Of the first class, then, or *positively perfect cures*, we have 4 occurring within the thirtieth day; 4 between the thirtieth and fortieth; 7 between the fortieth and fiftieth; 2 between the fiftieth and sixtieth; 5 between the sixtieth and eightieth; 5 between the eightieth and the one hundred and tenth: and 1—such, alas, is the occasional tardiness of cure—not sooner than the one hundred and seventy-first. Of the second class, or *virtually perfect* cures, 5 occur between the tenth and thirtieth day; 3 between the thirtieth and seventy-seventh day: total 8. Grand total, 36 cures.

The case not included in either class is case twenty-three, the only one in which it is positively stated that the disease was in the last stage, as evinced by "dulness over all the superior regions of the chest," and "a cavernous *râle*, accompanied with perfect pectoriloquy, below the centre of the left clavicle." This case, however, although *not stated in words* to be cured, tends by no means to damage the glory of Dr. John Hastings and his matchless specific, as the following short extract will show:

> "After a lapse of *sixteen days*, the cough, expectoration, and difficulty of breathing had diminished, the night-perspirations and other signs of hectic fever *had disappeared for rather more than a week*, the appetite was natural [it had previously been bad], and a dry blowing respiration was well marked over the space occupied by the cavern. The dose of naphtha was now increased to twenty drops. On the twentieth day he had a return of all his former symptoms, owing to his getting wet and allowing his clothes to dry on him. *The cavern, nevertheless, below the clavicle could hardly be detected.* . . . By the thirty-fourth day, a further amendment had evidently ensued, *for percussion yielded a much clearer sound*, and the respiratory murmur was improved. The cavernous *râle* was less distinct and *pectoriloquy* could not be detected. *Percussion elicited a good sound throughout the chest.*"!! (p. 89.)

In justice to Dr. John Hastings and ourselves, we will extract two or three of his perfectly-cured cases, selecting those which will occupy least space. We shall give them *verbatim* and complete.

"Case xi. Thomas Howell, a married man, aged twenty-two years, residing at 16, Foley street, Foley place, of a consumptive family, his father, brother, and five other members of his family having died during the last twelve months from pulmonary consumption, and a sister being now confined to her bed from the same cause, consulted me for the first time, on the twenty-fourth of March, 1843. He was following the trade of a shoemaker. About two months ago he caught cold while riding at night outside a coach from Cheltenham to London. This was followed by cough, which ceased in the course of a week; leaving, however, a little shortness of breath. The cough returned a fortnight ago, without any apparent cause, which had daily grown worse, and a rapid loss of flesh and considerable debility was the direct consequence. His general health, which had always been excellent, remained unimpaired, and the only other complaint he had to make, was a little dimness of vision which he had suffered from more or less since childhood. The chest was well formed, although its motions were somewhat restricted, and the clavicles were unusually bent outwards. A dull sound was the result of percussion over all the superior regions of the chest, and the respiratory murmur was generally feeble and harsh over the same spaces. The sounds of the heart were very distinct below the right clavicle. After taking naphtha for seven days, the cough had ceased to trouble him, and the sounds of the heart were scarcely audible below the right clavicle. At the end of fourteen days he had no complaint to make, and was growing stouter and stronger; percussion yielded a clear sound over the whole of the upper part of the chest, the respiratory murmur was much improved, and the sounds of the heart were inaudible below the clavicle. On the twenty-seventh of April, being the thirtieth day of the naphtha treatment, he enjoyed excellent health. Percussion continued to yield a natural sound, and the respiratory murmur was slightly harsh below the left clavicle." (pp. 71-3.)

"Case xvi. Eliza Shepherd, a married woman, aged forty-one, residing at 39, Carnaby street, Regent street, consulted me on the eleventh of May, 1843. Her father and several branches of his family had died through consumption. For some time, she had been troubled with cough and expectoration attended with difficulty of breathing and nocturnal perspirations, and during the last six weeks had greatly fallen away. Her pulse was eighty and weak, her bowels constipated; there was great loss of appetite, and a sensation of pain at the pit of the stomach after meals. The *catamenia* was regular. The walls of the chest were very much attenuated, there existed considerable depressions in the neighbourhood of both clavicles, and on the left side the movement of expansion was hardly perceptible. There was a dulness of sound over all the upper regions of the chest, and the respiratory murmur was generally feeble and harsh, the sounds of the heart being very distinct on the right side. In order to correct the bowels two aperient pills occasionally at bed time were prescribed, and the naphtha treatment in fifteen drop doses commenced. The night perspirations soon ceased. At the end of seven days the cough and expectoration had diminished, and the appetite had improved, but she complained of pain between her shoulders. The drops were now increased to twenty, and a mustard poultice applied between the shoulders. At the close of the fourteenth day she had greatly benefitted in every respect and possessed a good appetite. On the twenty-eighth day the cough, expectoration, and difficulty of breathing had entirely left her, and the sounds both from percussion and auscultation were of a healthy character." (pp. 79-80.)

"Case xix. Margaret Lee, a married woman, aged twenty-seven years, of 47, Stanhope street, Clare Market, informed me, on her admission as a patient on the fourth of May, 1843, that as far as her knowledge of her family history extended, the only member of her family who had died from consumption was her brother. For the whole of the past winter she had cough, expectoration, difficulty of breathing, and nocturnal perspirations, and for the last month had rapidly wasted and lost strength. The *catamenia* and the bowels were natural, the appetite was deficient, and some pain at the pit of the stomach was experienced after meals, attended with flatulence and palpitation. A dull sound was generally the result of percussion over the superior regions of the chest, and the respiratory murmur was then feeble and harsh, while below the right clavicle a dry crackling *râle* was distinct. The heart sounds were loud over the right side. The fourteenth day having arrived, after taking fifteen drops of naphtha in the usual quantity of water, the cough and perspirations had subsided, the difficulty of breathing had diminished, the appetite had improved, and the signs from percussion and auscultation were now satisfactory. The dose of naphtha was increased to twenty drops. In the course of the thirtieth day after the employment of the naphtha, the symptoms enumerated were removed, and the chest sounds and respiratory murmur were of a healthy nature." (pp. 83-4.)

Now is not this—and all this—most wonderful? Are not the results here blazoned almost unexampled in the annals of medicine, even when we include therein those copious but disgraceful and degrading chronicles of which our newspapers are the vehicles and of which the vendors of secret pills and balsams and other *universal* remedies, are the authors? We think they are. Other wonder-workers in the therapeutics of desperate maladies have for the most part toiled in the dark. In regard to the dreadful diseases they boasted to cure, honest men had the consolation to think, amid their own failures, that the diseases either did not exist at all, or that the so-called cures were merely temporary alleviations, to which almost all diseases are naturally subject. No evidence but the assertions of the parties was adduced; and, of course, these went for nothing among men of sense and honour. But the deeds of Dr. John Hastings are of quite another stamp. Wisely judging, no doubt, that the mere assertion of a doctor of medicine, even although that doctor should be the senior physician of a free dispensary, would, on such a subject, go for little, even among the well-informed men out of the profession,—now, when the knowledge of auscultation has become so general, —he has called to his aid *the positive evidence of physical signs*, leaving no loophole whereby the most sceptical (admitting the truth of the statements) can escape from the conviction that he has actually cured, in succession,* at least six and thirty cases of confirmed phthisis pulmonalis (and God knows how many more,) with consolidation of the whole or greater portion of the upper lobes, in one, two, or three months,—and all by some ten or twenty drops of naphtha taken thrice daily in a little water!! This is a feat assuredly "beyond all Greek, beyond all Roman fame;" beyond all that has been claimed for their panaceas by the quacks of past or present times; beyond all that has been observed by faithful and experienced physicians since the days of Hippocrates; beyond all that the most enthusiastic believers in the remedial powers of art have ever ventured to expect; beyond all that the fondest lovers of their kind have dared to hope or almost to pray for!

And can we, then, sitting here as capable, honest, and unprejudiced

* Of the thirty-seven cases, all, except four, occurred during the first six months of the present year—viz. four in January, four in February, six in March, eight in April, seven in May, and four in June. We should like to know if any other physician to a *general* dispensary, or even to a dispensary devoted exclusively to pectoral complaints, ever before met with so many cases of *chests dull all over the upper regions*, in the earlier stages of phthisis, in so short a period? We wish Dr. Roe or Dr. Walshe would inform us how many cases of this kind they have seen during the present year at the Hospital for Consumption. We wish still more earnestly that they and their colleague Dr. Williams would invite Dr. John Hastings to exhibit the Herculean heroism of his naphtha before them at the hospital. We dare say they could find, at least, a few specimens of disease for him, answering—or almost answering—to his stereotyped nosological formula:—"All the superior regions of the chest yielded a dull sound on percussion; the respiratory murmur was scarcely audible or was *feeble and harsh*, &c."

judges, lay this precious and flattering unction to our souls, and believe that all this has verily been done; and that we may now, in consequence, confidently expect, with Dr. John Hastings, to cure, by means of this glorious naphtha, every case or almost every case of pulmonary consumption, and thus, for the future, save from a premature death one fifth of the human race? Would that it were so; but alas, alas, it is not so! All our knowledge of health and disease, all our experience of medical treatment, all our reasoning on the facts of science, all our reflection on the fallaciousness of human testimony, on the imperfection of medical observation, on the sins of partiality and prejudice that so easily beset us, and on the seductions of vanity and self-interest, would lead us, *à priori*, to doubt or disbelieve such a proposition, if stated to us in general terms; and the careful perusal of the work before us has left on our minds the painful certainty that, in the individual case of Dr. John Hastings, we must utterly deny its truth!

The unexpected length to which this article has already run must prevent us from giving, in detail, all our reasons for coming to this distressing conclusion; and we must request such of our readers as have confidence in our judgment and good faith, to take its accuracy upon trust, until such time as they themselves have read the book *or tried the remedy*. We may, however, here put down, as briefly as possible, a few of the grounds on which our conclusion is based:

1. The extreme improbability that such a statement should be true, for reasons already stated as well as others.

2. The much greater likelihood that *any man* should be mistaken, than that events of such a marvellous character should have really taken place.

3. The infinitely greater likelihood that a man of the intellectual caliber and degree of education of Dr. John Hastings (as shown by his book) should be mistaken, than that the alleged facts should be really facts.

4. The general character of the cases as detailed in the book render it extremely probable that such a mistake has actually taken place. Indeed, we are certain that no experienced auscultator will read these cases consecutively, and not come to the conclusion that, in their construction, the modesty of nature has been overstepped—that they are, in fact, not true transcripts of actual disease. They are so very perfect specimens; the physical signs are so very strong, so very precise, so charmingly and facilely responding to the presumed organic lesion, and so exactly alike in every case; they are so uniformly terrible in their aspect and yet so uniformly amenable to the remedy, so ostentatiously consistent and yet every here and there betraying so many little incongruities, that the old observer of old nature feels, when reading them, that he is not on his ancient ground, or rather that his ancient ground has been metamorphosed into something new,

> "And starts—for life is wanting there."

Do we then (it will be asked) presume to state or to insinuate that the cases here narrated were not actually seen by Dr. John Hastings, or that he has, in any respect, promulgated what he does not believe to be true? By no means: on the contrary, we have not the slightest doubt that every case here related is a *bona fide* case, and that every patient is to be found or heard of at the localities so carefully indicated: we believe that he imagined, and still imagines, that he heard and saw everything he describes, while examining his patients; and that, in drawing up the details of their cases, he intended to delineate, and thought he delineated, only what his senses communicated to him. We think it right, however, that he should be made aware that many of his medical brethren do not put so liberal a construction on his conduct and motives in composing and publishing his book. The quality of its materials, its tone and manner, its "taking" title, and the way in which it has been blazoned in the newspapers, to an extent far beyond the ordinary range of honest medical advertisement for the mere sale of books—all too forcibly remind them of the publication of works, the subsequent conduct of whose authors left no room for indulging the charitable belief that their productions were merely the offspring of ignorance and folly. The speedy lapse of these men into the practice of open and unblushing quackery, made it evident that they had calculated beforehand the effect that would be made on the public mind by their bold announcement—that a most destructive malady, usually deemed irremediable, was really "curable," and not only so, but that it had been "successfully treated" by themselves. Wherefore it is that honorable men doubt of Dr. John Hastings, and that we deem it our duty to point out, at least, the danger that he runs.

As we have said, we acquit Dr. John Hastings of all intention to deceive others; we believe he was himself deceived. He thinks he has been stating the truth; we believe that he has been egregiously mistaken. And all this is readily explicable, and indeed is easily explained. Grant that Dr. John Hastings possesses a sanguine temperament and a lively fancy; that he has never been taught the difficult art of observation; that his mind is by nature ill qualified for, or has been ill disciplined in the processes of logical reasoning, and ill instructed in the laws of evidence; that he is an indifferent pathologist and an inexperienced auscultator,—and we need have no difficulty in accounting for and explaining all the marvels of his book, and reconciling them all with our conclusions.

Such is our conscientious judgment of the book of Dr. John Hastings, and such our theory of the marvels it unfolds. We believe the one to be just and accurate, and we think the other sufficient. Both, however, *may* be wrong. It is certainly *possible* that, in the cases narrated, the physical signs were accurately perceived and correctly delineated, and that they were, as in other instances, faithful interpreters of the presence of organic changes in the lungs; it is equally *possible* that, in the same cases, the naphtha taken by the patients may have, in the wonderfully brief space of a few weeks, thawed and resolved into a dew those vast and solid masses of tubercular deposit, as the sun in April melts the snow-wreaths of winter. It becomes us, therefore, to add—and in doing so we shall conclude this article, already too long—that it is our intention to give a fair and full trial of naphtha, in our own practice, and to urge our friends, as we now urge our readers at large, to do the same; and we hereby promise that if we shall ourselves observe even an approach to the success recorded in this book as flowing from it; or if we shall receive from competent and honest judges the assurance that they have been so fortunate, we shall not merely make public the results, but shall, as openly as we have accused Dr. John Hastings, avow our own mistakes and false judgment, and claim his forgiveness.

